# “Needed but lacked”: Exploring demand- and supply-side determinants of access to cardiopulmonary resuscitation training for the lay public in China

**DOI:** 10.3389/fpubh.2023.1164744

**Published:** 2023-04-12

**Authors:** Xuejie Dong, So Yeon Joyce Kong, Hanbing Xu, Andrew Fu Wah Ho, Audrey L. Blewer, Tonje Soraas Birkenes, Helge Myklebust, Xiaojian Zheng, Minghua Li, Zhi-Jie Zheng, Zhifeng Zhang, Lin Zhang

**Affiliations:** ^1^Department of Global Health, School of Public Health, Peking University, Beijing, China; ^2^Laerdal Medical Cooperation, Stavanger, Norway; ^3^School of Public Health, Shanghai Jiao Tong University, Shanghai, China; ^4^Department of Emergency Medicine, Singapore General Hospital, Singapore, Singapore; ^5^Pre-Hospital and Emergency Research Centre, Duke-National University of Singapore Medical School, Singapore, Singapore; ^6^Centre for Population Health Research and Implementation, SingHealth Regional Health System, Singapore, Singapore; ^7^Department of Family Medicine and Community Health, School of Medicine, Duke University, Durham, NC, United States; ^8^Department of Population Health Sciences, School of Medicine, Duke University, Durham, NC, United States; ^9^Shanghai Medical Emergency Center, Shanghai, China; ^10^School of Nursing, Shanghai Jiao Tong University, Shanghai, China

**Keywords:** cardiopulmonary resuscitation, lay public training, access, demand, supply

## Abstract

**Background:**

Despite years of public cardiopulmonary resuscitation (CPR) training efforts, the training rate and survival following out-of-hospital cardiac arrest (OHCA) have increased modestly in China. Access is imperative to increase the public CPR training rate, which is determined by both demand- (e.g., the lay public) and supply-side (e.g., CPR trainers) factors. We aimed to explore the demand and supply determinants of access to CPR training for the lay public in China.

**Methods:**

Qualitative semi-structured interviews were conducted with 77 laypeople (demand side) and eight key stakeholders from CPR training institutions (supply side) in Shanghai, China. The interview guide was informed by Levesque et al. healthcare access framework. Data were transcribed, quantified, described, and analyzed through thematic content analysis.

**Results:**

On the demand side, the laypeople's ability to perceive their need and willingness for CPR training was strong. However, they failed to access CPR training mainly due to the lack of information on where to get trained. Overestimation of skills, optimism bias, and misconceptions impeded laypeople from attending training. On the supply side, trainers were able to meet the needs of the trainees with existing resources, but they relied on participants who actively sought out and registered for training and lacked an understanding of the needs of the public for marketing and encouraging participation in the training.

**Conclusion:**

Insufficient information and lack of initiative on the demand side, lack of motivation, and understanding of public needs on the supply side all contributed to the persistently low CPR training rate in China. Suppliers should integrate resources, take the initiative to increase the CPR training rate, innovate training modes, expand correct publicity, and establish whole-process management of training programs.

## 1. Introduction

Out-of-hospital cardiac arrest (OHCA) represents a major global health problem with varied survival among communities and regions (1). Bystander cardiopulmonary resuscitation (CPR) is crucial for saving OHCA patients. The chances of survival increase 2- to 3-fold when CPR is performed by lay rescuers before emergency medical services (EMS) arrive (2). CPR training for the lay public is important to improve bystander CPR rates and for bystanders to deliver high-quality CPR (3, 4). It is estimated that a community CPR training program may reduce the OHCA mortality rate by 7.5% (5). Unfortunately, bystander CPR rates and CPR training rates have remained low in many regions, particularly in low- and middle-income countries (6, 7).

In China, CPR training programs for the lay public were first introduced in the mid-1980s and are now available through organizations such as the Red Cross, EMS centers, hospitals, community health centers, medical rescue associations, medical schools, commercial training institutions such as the American Heart Association (AHA), and public welfare organizations (8). With the launch of the Healthy China Initiative, the progressively expanding Public Access Defibrillator (PAD) programs and the growth of public attention on first aid, public awareness of and demand for CPR training is increasing (9). The training programs and institutions are mostly concentrated in east China, where there is a high level of economic development (10). However, huge challenges remain: The rate of public CPR training, bystander CPR rates, and OHCA survival are all < 1% in China (10).

Access to CPR training programs is imperative to increase the rate of public CPR training (11). Common theories have defined access as the opportunity to reach and obtain appropriate healthcare services in situations of perceived need for care (12, 13). According to the framework of access developed by Levesque et al. (14), access could be viewed as the possibility to identify healthcare needs, to seek healthcare services, to reach healthcare resources, to obtain or use healthcare services, and to be offered services appropriate to the needs for care. Concerns about access to healthcare (in this case, CPR training) must take into account both demand- and supply-side determinants. However, research and policies on CPR training are mainly related to the supply-side, such as training strategies, methodologies, contents, population-targeted courses, and refresher training sessions (15–18), whereas less focus has been given to demand-side features. To our knowledge, the only studies that have been carried out on the demand side have either described the CPR training rate of whole populations or just focused on a trained population (skill acquisition and retention, satisfaction, and willingness) (19–22). Hence, there is a lack of understanding of the “non-users” of CPR training services (23).

Before suggesting a strategy to improve access to CPR training for the lay public, it is imperative to characterize the barriers to the lay public to utilize CPR training. This study aimed to explore the demand- and supply-side determinatnts of access to CPR training for the lay public in China utilizing the access to the healthcare framework.

## 2. Materials and methods

### 2.1. Study design and setting

From January to July 2020, we conducted interviews with the lay public (demand side) and key informant stakeholders from institutions delivering CPR training for the lay public (supply side) in the Huangpu district of Shanghai. The main types of CPR training institutions in this district include the Red Cross, EMS centers, commercial training institutions (e.g., the AHA), and medical school-based training organizations. The study received ethics approval from the Joint Research Ethics Board of the Shanghai Jiao Tong University Schools of Public Health and Nursing (SJUPN-202014). All respondents joined this study without compensation and provided their written informed consent before the interviews.

### 2.2. Subject and sample

For the demand-side interview, we worked with a student first-aid organization at Shanghai Jiao Tong University, started by interviewing family members and friends of the organization's members, and included more participants by snowballing. We used a purposive recruitment strategy to obtain a sample that included men and women of different age groups with varying occupations (medical-related or not) living in the Huangpu district. We took a stepwise process: firstly, we recruited 62 participants from January to March 2020 and conducted a preliminary analysis; to reach data saturation, we then included 15 more participants and found no new themes, ideas, or opinions.

For the supply-side interview, we invited directors from four training institutions located in the Huangpu district (the Red Cross, the EMS center, the AHA, and the medical school-based training organization). For each training institution, we interviewed the director and one trainer. As a result, we completed 77 demand-side interviews (D1–D77) and eight supply-side interviews (S1–S8).

### 2.3. Interview guide

**Figure 1:**
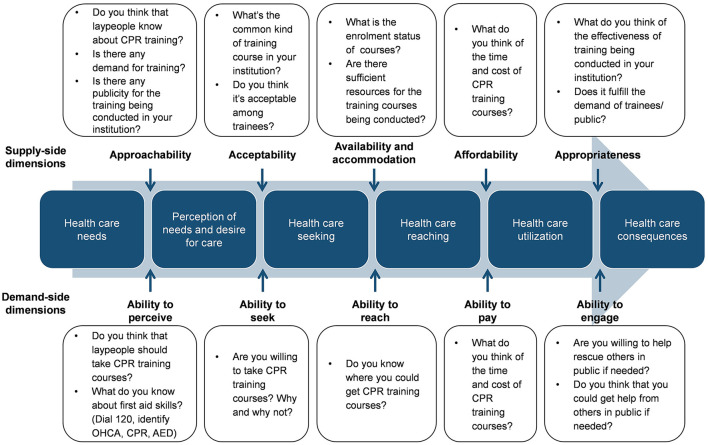
Key questions for demand- and supply-side interviews. CPR, cardiopulmonary resuscitation; 120, the universal number for emergency care and first aid in China; OHCA, out-of-hospital cardiac arrest; AED, automated external defibrillator. Reproduced from Levesque JF et al. licensed under CC BY 2.0, https://creativecommons.org/licenses/by/2.0/.

Qualitative semi-structured interviews were conducted individually either in-person or over the phone by trained interviewers in the Chinese language using interview guides designed for laypeople, directors, and trainers. The interview guide was informed by Levesque et al. (14) framework, which comprised five dimensions of attributes on the supply side: (1) approachability; (2) acceptability; (3) availability and accommodation; (4) affordability; and (5) appropriateness; and five dimensions of abilities on the demand side: (1) ability to perceive; (2) ability to seek; (3) ability to reach; (4) ability to pay; and (5) ability to engage (Figure 1) (14). Laypersons' demographic factors, including age, gender, education level, and CPR training history, were also asked. Each interview lasted 30–45 min, and all interviews were audio recorded.

### 2.4. Data collection and analysis

Qualitative content analysis was used to facilitate a deeper understanding of the interviewees' thoughts. The interviews were first replayed and transcribed, and then, meaningful statements by the interviewees were selected and coded independently by two researchers. Relevant themes identified were reorganized according to the demand and supply dimensions of the Levesque et al. (14) framework. Different answers were compared by searching for similarities and differences. Furthermore, the concepts and codes were grouped into patterns. The patterns were then ranked and listed in the order of frequency. In addition to descriptive statistics, illustrative quotations were presented to confirm the categories. Demand-side subjects were divided into groups according to their CPR training experience. Chi-square tests were used for comparisons between trained/untrained groups. Especially, in the ‘ability to perceive' dimension of the demand side, we asked subjects to convey their knowledge about first-aid skills. Differences between their self-rated and research-judged knowledge were compared using paired chi-square test.

## 3. Results

We completed 77 demand-side interviews and eight supply-side interviews (Table 1). For the demand side, most laypeople (76.6%, 59/77) had no previous CPR training history. Of the 18 trained respondents, only two attended paid courses. The findings are presented using the domains of access within the Levesque et al. (14) framework.

**Table 1:** Demographic characteristics of demand- and supply-side respondents.

**Demand-side: laypeople characteristics (*****n** =* **77)**	
**Female gender**, ***n*** **(%)**	41 (53.2)
**Age (years), mean±SD (range)**	38.8 ± 14.4 (15–72)
**Age group**, ***n*** **(%)**	
15–29 years	22 (28.6)
30–49 years	39 (50.6)
≥50 years	16 (20.8)
**Education level**, ***n*** **(%)**	
High school graduate or under	28 (36.4)
College graduate	42 (54.5)
Postgraduate or higher	7 (9.1)
**Occupation**, ***n*** **(%)**	
Non-medical-related	73 (94.8)
Medical-related	4 (5.2)
**CPR trained**, ***n*** **(%)**	
School/company organized free courses	16 (20.8)
Paid training courses	2 (2.6)
Untrained	59 (76.6)
**Supply-side: training providers' characteristics (*****n** =* **8)**	
**Female gender**, ***n*** **(%)**	3 (37.5)
**Age (years), mean±SD (range)**	38.6 ± 8.1 (29–49)
**Position**, ***n*** **(%)**	
Director	4 (50.0)
Trainer	4 (50.0)
**Years of working, mean±SD (range)**	11.4 ± 5.2 (4–90)
**Self-reported trained population**, ***n*** **(%)**	
< 1000 per year	2 (25.0)
2000–3000 per year	2 (25.0)
5000 per year	2 (25.0)
30000 per year	2 (25.0)

### 3.1. Perception of needs and desire for care

#### 3.1.1. Ability to perceive

Most laypeople (76/77, 98.7%) conveyed a clear understanding of the benefits of attending CPR training and believed that CPR training was needed using strong affirmations such as “*It's definitely needed!*” or “*Of course it is needed*.” Only one untrained participant saw no need for CPR training:

“*I think it (CPR) is the doctor's business, we rarely get into it...I often see people teaching CPR on TV and mobile phone, I think it's easy and I can learn from the internet by myself*.” (D24, untrained, male, 49, engineer, college graduate)

**Figure 2:**
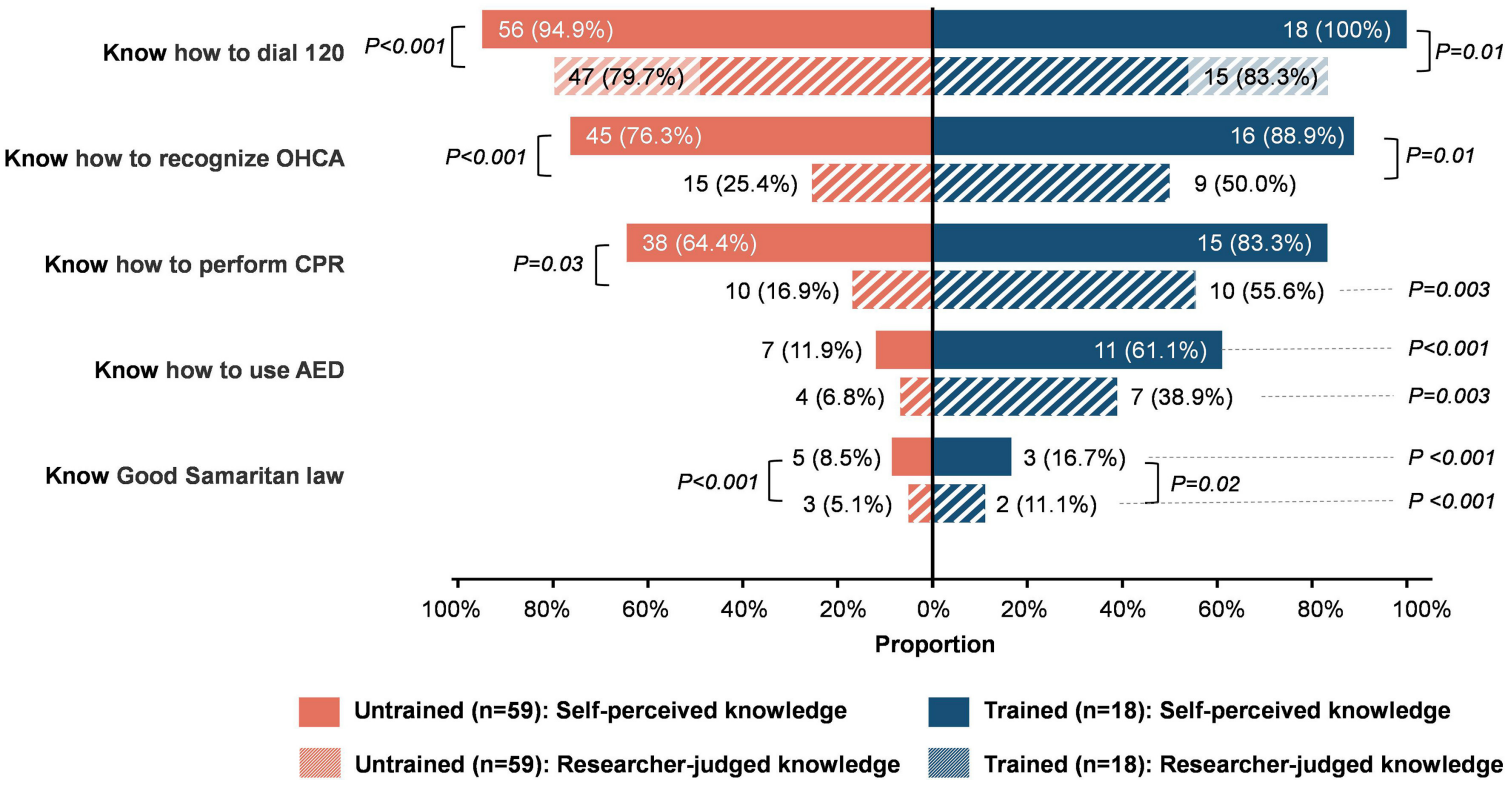
Demand-side respondents' self-perceived and researcher-judged first-aid knowledge. Values are shown as proportions. 120, the universal number for emergency care and first aid in China; OHCA, out-of-hospital cardiac arrest; CPR, cardiopulmonary resuscitation; AED, automated external defibrillator.

Laypeople generally overestimated their knowledge of first aid. Although the trained participants knew much more than the untrained, their researcher-judged knowledge was still poor (Figure 2). Many respondents answered that they “*know how to perform CPR*,” but there were many errors in their descriptions:

“*(To do CPR) you need to check the patient's breath, if no breathing, use your hand to press his/her chest, above the heart, press about 30 times a minute*.” (D48, untrained, female, 35, primary school teacher, college graduate)

“*It's too professional for me to describe it*.” (D15, untrained, male, 49, manager, college graduate)

“*I've been trained to do CPR. The basic gesture is to put one hand on the other, place hands on the left side of patient's chest, press the chest about 50 times a minute. The compression should be deep enough to be effective, at about 3 cm. After 10 compressions, you need to do 1 mouth-to-mouth ventilation*.” (D19, trained, male, 48, salesman, college graduate)

#### 3.1.2. Approachability

There was consensus among training providers that the public was not skilled in CPR and lacked the understanding or interest regarding CPR training. They admitted that most trainees registered for training on their own, and the feedback they got from these trainees could not fully reflect the public's idea. As for publicity, providers mainly relied on the website of their own institutions and partner organizations to promote and publicize training courses, with little commercial publicity. They thought that the publicity was quite effective since laypeople were coming to the training, and the volume of training had been relatively stable.

### 3.2. Healthcare seeking

#### 3.2.1. Ability to seek

Most laypeople (93.5%, 72/77) wanted to take CPR training, which included 100% (18/18) of trained respondents and 91.5% (54/59) of the untrained. Five untrained laypeople were reluctant to learn CPR, citing “*not my business*” (*n* = 3), “*lack of time*” (*n* = 1), and “*old age*” (*n* = 1):

“*I just don't feel like CPR is something that usually comes up around me, plus I don't have the time.”* (D15, untrained, male, 49, manager, college graduate)

“*I think this (CPR) is the doctor's business, we rarely get into it...”* (D24, untrained, male, 49, engineer, college graduate)

“*How can you think of learning it when nothing could happen? It's quite troublesome, and it's not worth paid for.”* (D12, untrained, female, 52, unemployed, primary school graduate)

“*I don't have the time or interest to learn.”* (D35, untrained, 72, female, retired, middle school graduated)

“*I'm too old to learn it, and I don't have much strength.”* (D54, untrained, female, 72, retired, primary school graduate)

For the paradox of laypeople wanting to learn CPR but not attending any courses, 54 participants provided three main reasons, including “*don't know where to learn*” (*n* = 26, 52.5%), “*no opportunity/ have not been offered such courses*” (*n* = 15, 25.5%), and “*not my business*” (*n* = 13, 22.0%), and the typical responses included the following:

“*I think CPR training is needed, and I want to learn it very much, but I just don't know where to get trained. Maybe the publicity is not enough, or because our community and industry don't have this kind of training site.”* (D3, untrained, female, 45, waiter, high school graduate)

“*Because CPR training is not mandatory, there is no opportunity, no one have taught it, communities or companies have not held these activities.”* (D51, untrained, male, 47, businessman, high school graduate)

“*I have never encountered similar situations that need CPR, and I think it's impossible for me or my family to be in such situation.”* (D1, untrained, female, 51, sales, college graduate)

Some respondents even mentioned female gender and old age to be “*inappropriate*” or “*have no chance*” to learn CPR:

“*I was told by the doctor of our factory that it required much strength to do CPR, to move patient or do other operations, so in principle, the factory usually make men worker to be the first-aid member, supposing that women are not very suitable.”* (D27, untrained, female, 48, engineer, college graduate)

“*I didn't think it was important to learn (when I was young). Now I get older, I get more health awareness, I think it's necessary but I don't have the chance anymore.”* (D16, untrained, female, 46, middle school teacher, college graduate)

#### 3.2.2. Acceptability

According to training providers, they mainly conducted the courses in their own institutions, with occasional activity-based training in communities, schools, or companies. The existing courses had no restrictions on age, gender, or occupation. Their training contained theoretical lectures and hands-on practice content covering chest compression, mouth-to-mouth ventilation, AED use, and the Good Samaritan Law. Their trainees were satisfied with the training, and although they might have hygiene concerns about mouth-to-mouth ventilation, they were still willing to participate. Other than their requirements to obtain a first-aid certificate, few trainees were concerned with the type of institution or the level of accreditation.

### 3.3. Healthcare reaching

#### 3.3.1. Ability to reach

**Figure 3:**
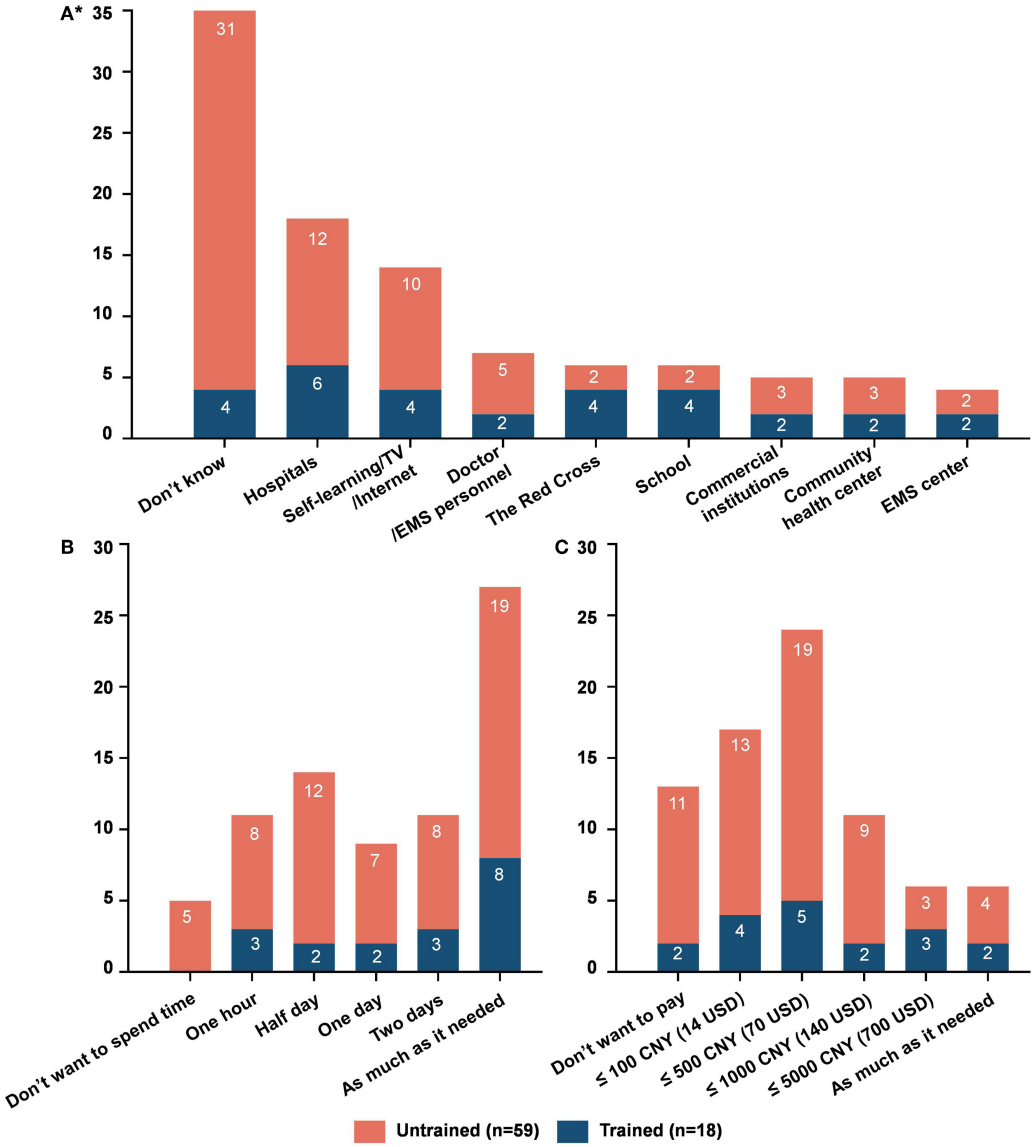
Demand-side respondents' perceptions of where to get CPR training **(A)**, and willingness to spend time **(B)** and money **(C)** on CPR training courses. *means that multiple responses were allowed for question A. Values are shown as exact numbers. EMS, emergency medical services; CNY, China Yuan (1 CNY ≈ 0.14 USD).

Laypeople reported their confusion about where to learn CPR. While CPR training could now be accessed in many ways, nearly half of the participants (45.5%, 35/77) did not know where to get CPR training, and their opinions varied (Figure 3A). Some believed that “…*people must go to designated places to get training*,” while a considerable proportion believed that self-learning could be done through the TV or the internet.

“*There must be a lot of TV programs and popular science posts on the Internet, we could learn by watching them.”* (D30, trained, female, 19, college student)

“*I believe there are formal online teaching websites that have such training contents.”* (D19, trained, male, 48, salesman, college graduate)

#### 3.3.2. Availability and accommodation

According to trainers, the source of trainees was laypeople who signed up through publicly accessible platforms and each session was close to capacity. There were also times when training activities were organized by the community/company for the participation of its personnel. The training providers considered the resources for training to be adequate, including premises, equipment, personnel, funding, and policy support. Regarding online vs. offline courses, most reported offering offline training (e.g., in-person), with hands-on practice to ensure effectiveness. There were also online courses, but instructors did not consider them as effective as the offline courses.

### 3.4. Healthcare utilization

#### 3.4.1. Ability to pay

Laypeople expressed varied opinions about the time and money spent on training courses (Figures 3B, C). Concerning the time required, except for five respondents who expressed no willingness, all were willing to take time and had strong flexibility from 1 h to 2 days, and 35.1% (27/77) thought it was fine to spend as much time as needed. In terms of cost, 16.9% (13/77) were unwilling to spend any money, most were willing to pay up to 500 CNY (≈ 70 USD), and only 7.8% (6/77) were willing to pay as much as needed. Some respondents indicated a willingness to devote a large amount of time to the training but were not willing to pay much for it. Explanations they gave included “*CPR training should be free and universal for all”* and “*Free videos/ online courses are enough”* (Table 2).

**Table 2:** Demand-side respondents' varied views on the time and money spent on CPR training.

**# Participants**	**Opinion and verbatim quotes of time spent**	**Opinion and verbatim quotes of money spent**
D6, trained, male, 48, manager, college graduate	“*Anytime, as long as I learn how to do CPR.”*	“*I think this (CPR training) should be universal and free to everyone.”*
D16, untrained, female, 46, high school teacher, college graduate	“*No matter how long it takes.”*	“*… I think this (pay for training) is unacceptable.”*
D18, trained, male, 22, college school student	“*Maybe half an hour to one hour is enough.”*	“*It depends on the price. There's a lot of this stuff on the Internet, it should be free, this kind of…like general knowledge education, I think there should be a lot of free instructions. I will not choose courses that actually cost money.”*
D27, untrained, female, 48, engineer, college graduate	“*Half a day is OK.”*	“*I don't accept paid courses.”*
D29, untrained, male, 51, manager, college graduate	“*Half a day to 1 day, as long as it doesn't interfere with my work.”*	“*(long pause) … This (to pay money) seems not too much necessary, I am not a professional.”*
D40, untrained, female, 20, college school student	“*Complete in 1 day.”*	“*No, I can't.”*
D57, untrained, female, 32, sales, high school graduate	“*It depends on the content. I'll cooperate when I have time.”*	“*This is common knowledge, so I don't feel very need to spend money on that.”*
D59, untrained, male, 19, college school student	“*No more than 1 h each time. If the teaching is very comprehensive and systematic, I am willing to spend more time.”*	“*Not quite willing to pay for it.”*
D68, trained, female, 50, teacher, postgraduate	“*One day is fine.”*	“*I don't want to pay.”*
D71, untrained, female, 35, accountant, postgraduate	“*If there's an organization to give training, I'd like to take that time. If not, it's hard to pick my own time.”*	“*I may not accept to spend money. There are medical workers in my family, so I can learn from them.”*

#### 3.4.2. Affordability

According to training providers, the duration for existing courses was 1 h to half a day, with less time being perceived as insufficient for quality. The cost of courses ranged from free, 200–500 CNY (≈ 30–70 USD) to 1000–2000 CNY (≈ 140–280 USD) depending on the type of training institution. The trainers did not report any concerns from the trainees regarding the training duration or cost. For long-term consideration, due to the cost of the venue, equipment, and consumables, a free program cannot be offered, and the reasonable cost with quality assurance would be 100–300 CNY (≈ 14–42 USD).

### 3.5. Healthcare consequences

#### 3.5.1. Ability to engage

**Figure 4:**
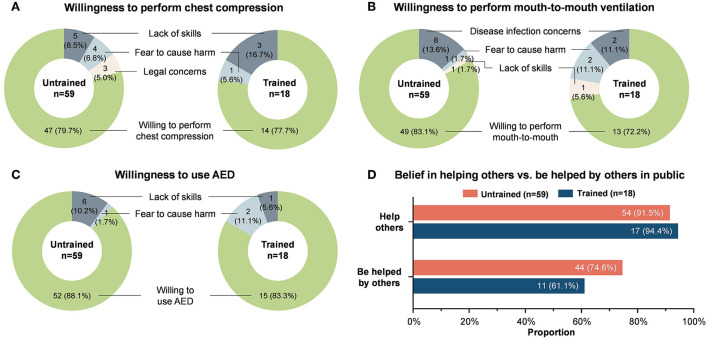
Respondents' willingness to perform chest compression **(A)**, mouth-to-mouth ventilation **(B)**, and AED **(C)** on others, and belief in helping others and being helped by others in public **(D)**. Values are shown as proportions. AED, automated external defibrillator.

Most laypeople were willing to perform chest compression (79.2%), mouth-to-mouth (80.5%), and use AED (87.0%) on individuals in need (Figures 4A–C). Reasons for unwillingness included “*Lack of skills*,” “*Fear of causing additional harm*,” “*Legal concerns,” and “diseases infection concerns*.” Most respondents were sure they would help strangers if needed in public places but were not sure they would receive help if the situation was reversed (Figure 4D, Table 3).

**Table 3:** Demand-side respondents' varied views on helping others and getting help from others in public.

**# Participants**	**Opinion and verbatim quotes of helping others**	**Opinion and verbatim quotes of getting help from others**
D10, trained, female, 50, manager, college graduate	“*I will help if I get skilled, if I don't have the skill, I would definitely find someone to help.”*	“*(hesitating)… It's not easy for me to force others to help me.”*
D13, untrained, female, 45, teacher, postgraduate	“*Yes of course, whatever it takes!”*	“*It depends… I'm not sure.”*
D16, untrained, female, 46, high school teacher, college graduate	“*(firmly) Yes I will.”*	“*(uncertainly) There should be someone can help.”*
D17, trained, male, 18, college student	“*Yes.”*	“*I think the probability is quite small, because the first aid knowledge has not been widely popularized.”*
D37, untrained, female, 25, teacher, college graduate	“*I will do what I can to help.”*	“*Not sure. Many people are deterred by the fear of public opinion.”*
D58, trained, male, 50, manager, college graduate	“*We are laypeople, we are there to help others. Even if I'm not confident, I will still help others.”*	“*I can't answer that question. I can only promise myself as a citizen to help others on moral grounds. I don't know about others.”*
D61, trained, female, 19, college student	“*Yes, I'm not much confident, but I'll try my best.”*	“*It depends on whether there are good people around. It really depends on luck. Sometimes you may meet people who understand those skills, and sometimes you may not.”*
D70, trained, female, 35, teacher, postgraduate	“*I will try to help, but I'm afraid to operate first-aid by myself.”*	“*Consider the fear of myself, I don't think there are many people who dare to help me.”*

#### 3.5.2. Appropriateness

Training providers felt that existing courses were effective and trainees were satisfied with the training received, but due to a lack of good registry and following-up, they were not sure that trainees would use their gained skills in real emergencies. Trainers thought their courses fulfilled the needs of the trainees because they felt that trainees had high levels of skill acquisition and satisfaction. The annual number of trainees of the interviewed institutions was in line with the carrying capacity of the institution (funding, personnel, space, and frequency), and providers believed that expanding the existing model could gradually increase the overall training rate of the local region.

## 4. Discussion

This study adopted the Levesque et al. (14) framework to explore the demand and supply determinants of access to CPR training for the lay public in China. We found that the ability of the demand side to perceive their need and willingness for CPR training was strong, but they lacked the ability to reach CPR training, mainly because they did not know where to get trained. Unlike previous surveys in which a lack of awareness of the need impeded laypeople seeking training (24–26), our respondents showed a strong understanding of the need for training, which gave us a chance to get a deeper understanding of the “non-users” of CPR training services. Overestimation of skills, optimistic bias, misconceptions, and concerns were common among laypeople, which negatively impacted their willingness to attend training. On the supply side, training providers were able to meet the needs of the trainees with existing resources, but they relied on participants who actively sought out and registered for training and lacked an understanding of the needs of the public for marketing and encouragement to participate in the training.

### 4.1. Demand-side determinants of access to CPR training

Laypeople's lack of information on where to get training, and the belief that CPR skills could be acquired by self-learning impeded their CPR training attendance. Like other studies, legal issues, inadequate knowledge, and fear of infection were the main reasons for laypeople's unwillingness to help others (9, 24), and the idea that female participants and seniors were physically incapable of performing or learning CPR also hampered their willingness (25–27).

The trained respondents had a higher level of first-aid knowledge and more awareness of CPR training than the untrained, yet a noteworthy finding was that both groups were quite close in their responses to all of our other questions. On the one hand, it could indicate a poor outcome of current training regarding skill acquisition in that participants are not more willing to use the skill obtained, which could be due to the varied quality of training programs. On the other hand, it could mean that information on CPR and its training can be obtained by the lay public without training. Noticeably, the level of self-perceived CPR knowledge for trained and untrained groups is both higher than that reported in China years ago (9). This improvement might be related to enhanced publicity and popularization of science in recent years in China.

Many laypeople expressed “no opportunity/not been offered such courses,” which seemed contradictory to the trainers' claims that there was enough training offered. Laypeople lacked self-motivation to participate in CPR training: They expected the training to be mandatory, to be held by communities or companies, and to be free, which was also reported by other studies (9, 26). However, would they take the training if they were offered one for free? In view of respondents who mentioned “not my business” as a reason for not being trained, the answer may still be “no.” As the causal mechanism of access indicated (28), the “not my business” mindset and the overestimation of one's knowledge and skills of others can lead to unawareness of healthcare needs, inactive healthcare seeking, passive participation, or refusing healthcare.

Although laypeople's willingness to help others has increased compared to a study years ago (9), they were uncertain that they could get help from others, indicating insecurity and distrust in community first-aid capacity. One respondent even believed that bystander rescue behavior was not encouraged, saying “*There are some slogans about CPR on TV, which encourage people's first-aid behavior verbally, but what I see every day in the news is that people get in trouble for saving lives, which tells us the less trouble the better*.” This distrust, we believe, would in turn block the public's perception of the need and enthusiasm to learn CPR.

### 4.2. Supply-side determinants of access to CPR training

While culture and values affected how laypeople sought CPR training, these concerns and misunderstandings were not originated from themselves but from inadequate or incorrect information they perceived. Inadequate publicity, prejudice, wrong messages (e.g., female participants or seniors could not handle the tasks and mouth-to-mouth might cause infections), and the fact that the public was more concerned with legal disputes than bystander rescue behaviors all contributed to this situation.

Training providers lacked a valid way to fully understand the real needs of the public. The only feedback they got was from trainees who initiated taking the courses, showing a high level of effectiveness and satisfaction. Owing to the lack of follow-up of certified trainees, the providers were not sure whether their training could translate into real rescue action. Although the trainers were acutely aware of the low training participation rate, they were quite satisfied with the effectiveness of the courses. Under the constraints of existing funds, manpower, and equipment, there was less motivation for them to change the training delivery modes.

### 4.3. Key recommendations for improving access to CPR training for the lay public

Although it seems that supply exceeds demand, CPR training for the lay public is a program for the good of the public rather than a commercial product, and the basis of all discussions is that everyone should be trained in CPR. Currently, the only way for trainees to get CPR training is by seeking out and registering for training on their own. The large number of untrained respondents willing to learn CPR implied a crucial need for optimizing national CPR training programs. While there are two-side reasons, they all boil down to the supply side. To improve access to CPR training for the lay public, we propose the following key recommendations and areas of emphasis for the supply side:

First, training providers and policymakers should take the initiative to increase the CPR training rate. It is warranted to integrate existing training resources rather than each training institution doing its own work.

Second, innovative training modes of high efficiency and low cost should be advocated to improve the availability and affordability of CPR training. Strategies and policies to be taken into consideration include the following: introducing CPR education into school curricula, new employee orientation training, and driver's license training (29, 30); providing widespread availability of self-CPR training kits; and developing self-directed learning courses (31, 32).

Third, broader publicity of CPR and CPR training should be achieved. As we have discussed earlier, laypeople with low self-perceived demand for training might still refuse the opportunity of convenient and affordable CPR training. A policy must be combined with the right publicity to impact attitudes and eliminate the misconceptions and concerns of the lay public.

Fourth, a whole-process management of trainees and a uniform registry of rescue behaviors should be established. It is hoped that a culture of excellence could be formed through positive feedback from successful bystander CPR stories, and social insecurity could thereby be reduced.

Lastly, it is fundamental for training providers to have a clear and objective understanding of public demand and to keep in mind the match between demand and supply sides.

This study has several limitations. First, convenience sampling was conducted, thus the respondents may not be representative of the whole population. However, as qualitative research, the aim of this study was not to compute the training rate but to give in-depth views. Second, the interviews were conducted during the COVID-19 epidemic period, which may have a series of psychological effects on respondents, leading them to pay more attention to first aid or relevant information, and they may have more concerns than usual when indicating their willingness to help. These would both cause certain deviations. Third, the interviews have geographic limitations, and all the findings and discussions are related to a Chinese cultural and political context and may therefore not apply to other settings.

## 5. Conclusion

This study revealed the perceptions of access to CPR training for the lay public from both the supply and demand side utilizing the Levesque et al. (14) framework with a focus on “non-users” and provided an in-depth understanding of respondents' opinions. Plausible reasons for the low public CPR training rate in China include insufficient information, a lack of initiative on the demand side, and a lack of motivation and understanding of the needs of the public on the supply side. Training suppliers should integrate existing training resources, take the initiative to increase the CPR training rate, innovate training modes, expand correct publicity, and establish a whole-process management of their training programs.

## Data availability statement

The data that support the findings of this study are available from the corresponding author upon reasonable request.

## Ethics statement

The studies involving human participants were reviewed and approved by the Joint Research Ethics Board of the Shanghai Jiao Tong University Schools of Public Health and Nursing (SJUPN-202014). The patients/participants provided their written informed consent to participate in this study. Written informed consent was obtained from the individual(s) for the publication of any potentially identifiable images or data included in this article.

## Author contributions

XD, LZ, and ZZ contributed to the conception of the study. SK, TB, and HM contributed to the design of the study. XD, HX, XZ, and ML conducted the interviews and contributed to data acquisition. XD, SK, and HX conducted the data analysis. XD, SK, AH, AB, and TB contributed to the data interpretation. XD performed the drafting of the manuscript. SK, AH, AB, and TB finalized the manuscript. HM and Z-JZ provided administrative advice and consultations. All authors contributed substantially to the revision of the manuscript and approved the submitted version.
